# Development of Low-Cost Point-of-Care Technologies for Cervical Cancer Prevention Based on a Single-Board Computer

**DOI:** 10.1109/JTEHM.2020.2970694

**Published:** 2020-02-03

**Authors:** Sonia Parra, Eduardo Carranza, Jackson Coole, Brady Hunt, Chelsey Smith, Pelham Keahey, Mauricio Maza, Kathleen Schmeler, Rebecca Richards-Kortum

**Affiliations:** 1 Department of BioengineeringRice University 3990 HoustonTX77005USA; 2 Wellman Center for PhotomedicineHarvard Medical School and Massachusetts General HospitalBostonMA02114USA; 3 Basic Health International El SalvadorSan SalvadorCP1101El Salvador; 4 Department of Gynecologic Oncology and Reproductive MedicineThe University of Texas MD Anderson Cancer Center 4002 HoustonTX77030USA

**Keywords:** Cervical cancer prevention, low-cost medical technology, point-of-care, Raspberry Pi

## Abstract

Cervical cancer disproportionally affects women in low- and middle-income countries, in part due to the difficulty of implementing existing cervical cancer screening and diagnostic technologies in low-resource settings. Single-board computers offer a low-cost alternative to provide computational support for automated point-of-care technologies. Here we demonstrate two new devices for cervical cancer prevention that use a single-board computer: 1) a low-cost imaging system for real-time detection of cervical precancer and 2) a low-cost reader for real-time interpretation of lateral flow-based molecular tests to detect cervical cancer biomarkers. Using a Raspberry Pi computer to provide real-time image collection and processing, we developed: 1) a low-cost, portable high-resolution microendoscope system (PiHRME); and 2) a low-cost automatic lateral flow test reader (PiReader). The PiHRME acquired high-resolution ($4.4~\mu \text{m}$) images of the cervix at half the cost of existing high-resolution microendoscope systems; image analysis algorithms based on convolutional neural networks were implemented to provide real-time image interpretation. The PiReader acquired and analyzed images of a point-of-care human papillomavirus (HPV) serology test with the same contrast and accuracy as a standard flatbed high-resolution scanner coupled to a laptop computer, for less than one-fifth of the cost. Raspberry Pi single-board computers provide a low-cost means to implement point-of-care tools with automatic image analysis. This work demonstrates the promise of single-board computers to develop and translate low-cost, point-of-care technologies for use in low-resource settings.

## Graphical abstract

Cervical cancer disproportionally affects women in low- and middle-income countries, in part due to the difficulty of implementing existing cervical cancer screening and diagnostic technologies in low-resource settings. Here we demonstrate two new devices for cervical cancer prevention that use a single-board computer: 1) a low-cost imaging system for real-time detection of cervical precancer and 2) a low-cost reader for real-time interpretation of lateral flow-based molecular tests to detect cervical cancer biomarkers.

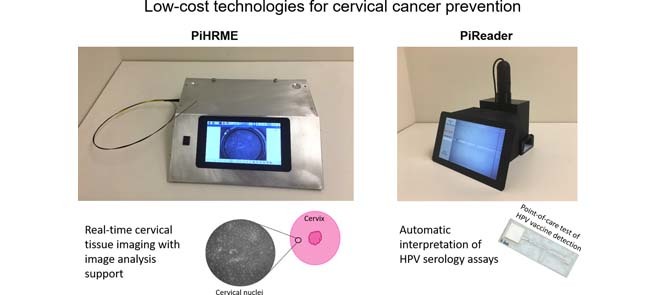

## I. Introduction

Cervical cancer continues to disproportionally affect women in low-resource settings. According to the most recent 2018 GLOBOCAN estimates, the incidence and mortality rate of cervical cancer in Low/Medium Human Development Index (HDI) regions are 18.2 per 100,000 and 12.0 per 100,000 respectively, nearly double the incidence rate and triple the mortality rate of that in High/Very High HDI regions [1]. One reason for this disparity is the difficulty of implementing existing cervical cancer prevention, screening, and detection technologies (e.g. HPV vaccination, Pap and HPV testing, and colposcopy) in low-resource settings [2]–[4].

To address this disparity, a number of point-of-care technologies to improve cervical cancer prevention, screening, and detection are in development [5]–[8]. Broadly, these strategies include: 1) new imaging tools to improve real-time detection of high-grade cervical precancer; and 2) new molecular assays for point-of-care detection of cervical cancer biomarkers.

A number of high-resolution imaging technologies have been developed to provide real-time detection of high-grade cervical precancer without the need for biopsy [9]–[11]. For example, the high-resolution microendoscope (HRME) is one low-cost technology that has been developed to provide in vivo imaging of the cervix at the point-of-care [12]–[14]. Image segmentation algorithms have been developed to characterize the size and shape of nuclei within the field-of-view, [8], [15], [16] demonstrating diagnostic performance on par with expert colposcopy for detecting high-grade cervical precancer and cancer [10]. However, these algorithms are often implemented on Windows PC systems that rely on proprietary and computationally heavy software frameworks (LabVIEW/MATLAB) and contribute significantly to the overall cost of the device. The latest version of the HRME system ($2,450) relies on a computer tablet, which accounts for 33% of the total cost.

Similarly, a number of lateral flow-based tests have been developed to detect biomarkers associated with cervical cancer [17]–[19]. Flatbed scanners are often used to capture and quantitatively analyze such tests, but these systems are not portable and require a computational interface [20]. Alternatively, lower-cost cell phone-based readers have been developed [21], [22], but it can be difficult to control parameters such as image gain for quantitative test interpretation, especially with rapid updates to cell phone operating systems that may affect image capture [23], [24].

Single-board computers, such as the Raspberry Pi^®^, have recently proven to be an effective way to reduce the cost and size of medical and scientific instruments, without sacrificing performance [25]–[29]. The low-cost and availability of open-source software frameworks make these computers a versatile tool in the development of point-of-care devices for use in low-resource settings.

Here we demonstrate the use of a Raspberry Pi single-board computer to integrate imaging and quantitative computational analysis in two low-cost, portable imaging systems for cervical cancer prevention. The first is a high-resolution imaging system with real-time analysis software and the second is a low-cost, portable lateral flow test reader to automatically read a point-of-care diagnostic test. Using a Raspberry Pi computer and open-source software frameworks, we reduced the cost of the HRME imaging system by half while maintaining image quality and still providing real-time image analysis support. Similarly, we used this platform to develop a low-cost reader for a lateral flow HPV serology test to assess whether a patient had received multiple doses of the HPV vaccine [30]; using a Raspberry Pi computer, we reduced the cost of the computer scanner system by 85% without sacrificing image contrast or accuracy.

## II. Methods and Procedures

### A. PiHRME

We developed and evaluated the PiHRME, a High-Resolution Microendoscope designed to capture and analyze high-resolution images of cervical epithelium in real-time; changes in nuclear morphometry are analyzed to detect the presence of high-grade cervical precancer.

#### 1) Hardware

**FIGURE 1.**
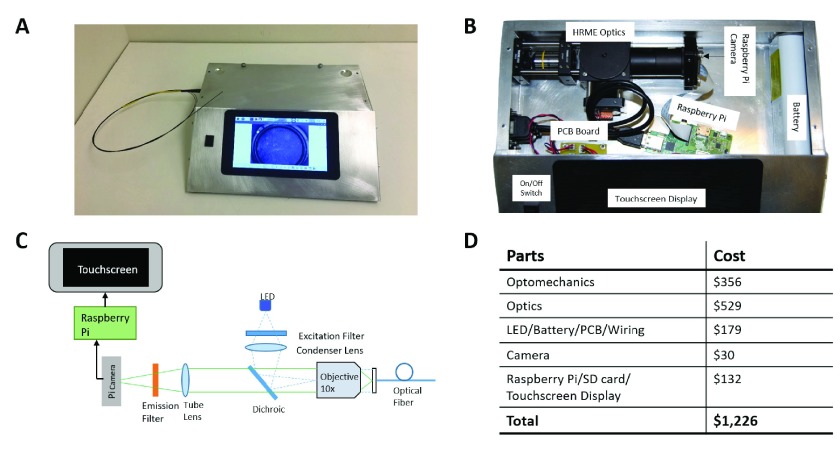
PiHRME system: A) device photo, B) device photo of inner components (labeled), C) schematic of the optical and computational components, and D) cost of materials.

**Fig. 1** shows a schematic diagram and photograph of the exterior and interior of the PiHRME, a portable fiberoptic fluorescence microscope system based on a Raspberry Pi computer. The optical setup was designed to match the optical performance of HRME systems previously described in Pierce *et al.* [31] and Quang *et al.* [16] with the main difference being that images were captured using a PiCamera sensor linked to a single-board Raspberry Pi computer rather than using a CCD camera sensor linked to a PC computer system. A high-power blue LED provides illumination at a center wavelength of 460 nm; illumination light is directed through a 475 nm shortpass filter and an aspheric condenser lens, which focuses the light on the back aperture of a $10\times$ objective (Olympus PlanC Achromat). Illumination light then propagates through a coherent optical fiber bundle (Fujikura Imaging Fiber, FIGH-30-850N) to illuminate the sample. The fluorescent signal emitted from the sample is captured by the same optical fiber bundle and objective. The emitted fluorescence then passes through a 485 nm longpass dichroic before being focused onto the Raspberry Pi v2 camera sensor (8 MP CMOS) by a tube lens (f = 60 mm). A 500 nm longpass filter is located immediately in front of the camera sensor to reject scattered light. The Raspberry Pi v2 camera is connected to a Raspberry Pi v3 computer board and integrated touchscreen display. The Raspberry Pi computer powers the camera and displays a live video feed via the touchscreen display. All images taken during an imaging session are saved on the Raspberry Pi computer in a folder identified by the date and time.

The optical system and Raspberry Pi computer are encased within a $30\times 25.5\times8$ cm aluminum enclosure which can easily fit within a backpack for transport. The entire system is powered by a 6.4 V lithium ion battery that is connected to both the LED driver and Raspberry Pi via a custom-designed PCB board. The main power switch automatically powers the LED and Raspberry Pi computer. The cost of material goods for the PiHRME was $1,226.

#### 2) Image Analysis

Previous versions of the HRME have incorporated image analysis software that relies on image segmentation to quantify changes in nuclear morphometry due to high-grade cervical precancer and early cancer [10], [15], [16]. This is consistent with current clinical guidelines for screening and treatment of cervical cancer and its precursors [32]. High-grade cervical precancer is at greater risk of progressing to cervical cancer than low-grade precancer or normal cervical tissue, therefore the early detection and treatment of high-grade precancer is important in preventing cervical cancer. Due to limitations in processing speed and availability of proprietary software frameworks on the Raspberry Pi, deployment of real-time image analysis using previously developed nuclei segmentation algorithms was not practical. As a proof of principle, real-time image analysis was accomplished on the PiHRME by employing a transfer learning approach to retrain a light weight convolutional neural network (CNN) (MobileNetV2) [33] to predict the output of the pre-existing HRME image analysis algorithm being validated in previous and ongoing clinical trials [10], [11].

A dataset consisting of 4,053 cervical images from an ongoing clinical study approved by the Institutional Review Boards at The University of Texas MD Anderson Cancer Center, Rice University, and Barretos Cancer Hospital in Brazil was organized with binary class labels (i.e. HRME positive or HRME negative) as defined by the pre-existing HRME algorithm. The class distribution of the dataset was 1,214 (30%) HRME positive and 2,839 (70%) HRME negative images. Open-source code for retraining TensorFlow models was then used to extract a feature vector of each image using the pre-trained network model to optimize the parameters of the final network layer to predict the new label (in this case HRME positive or HRME negative) [34]. Default parameters for learning rate (0.01), train/validation/test fractions (0.8/0.1/0.1 respectively), training batch size (100 images per batch), and number of training iterations (4000 training steps) were used. All model optimizations were performed on a desktop computer (Linux) using TensorFlow 1.11, after which final model parameters were saved to a disk and transferred to the PiHRME file system to be used for real-time inference on newly acquired images.

To evaluate the feasibility of using the model for real-time inference, the PiHRME frame rate was benchmarked with and without model inference on each frame. A simple user interface was developed to display the following information over each frame in the video feed on the PiHRME: image frame number, probability output of the CNN prediction (from 0 to 1.00), and speed of classification (reported in frames per second, FPS).

#### 3) In Vitro Evaluation

Image quality of the PiHRME was compared to the current tablet-based HRME system. Image resolution was determined by imaging a 1951 US Air Force Resolution Target. Image contrast was compared by plotting the intensity changes that occurred across the three vertical bars of Group 6 Element 1 of the same resolution target.

To evaluate the ability of the CNN algorithm to support real-time image analysis of images acquired with the PiHRME, we created and imaged fluorescent calibration targets that were representative of normal and precancerous cervical nuclear morphology. The calibration targets consist of small circular patterns (8–23 $ \mu \text{m}$ in size) against a black background that resemble cervical nuclei when imaging. While imaging these targets, the CNN image analysis algorithm was implemented so that a probability score appeared at the top of the touchscreen display and updated in real-time as new calibration targets were imaged.

#### 4) Ex Vivo Cervical Imaging

Ex vivo images of LEEP specimens (excised cervical tissue concerning for high-grade cervical precancer) were obtained using the PiHRME under a protocol approved by the Institutional Review Boards at The University of Texas MD Anderson Cancer Center and Rice University. Before imaging, LEEP specimens were stained with 0.01% proflavine solution, a topical antiseptic solution that has been used to fluorescently stain cervical nuclei for imaging, and Lugol’s iodine, to increase image contrast. Similar staining has been performed previously to acquire high-resolution images of the cervix using HRME imaging systems [10], [11]. All images were analyzed using the CNN image analysis algorithm.

#### 5) In Vivo Cervical Imaging

High-resolution in vivo imaging of the cervix using the PiHRME was performed in a low-resource setting in accordance with the protocol approved by the Comité Nacional de Ética de la Investigación en Salud (National Ethics Committee of Health Research) in El Salvador and the Institutional Review Boards at The University of Texas MD Anderson Cancer Center and Rice University. Women undergoing a routine colposcopy examination for cervical cancer prevention in El Salvador were imaged with the PiHRME. All visible lesions on the cervix along with one normal area were imaged and all visibly abnormal areas were biopsied. Before imaging, 0.01% proflavine solution and Lugol’s iodine were applied. Following image acquisition, images were analyzed using the CNN image analysis algorithm.

### B. PiReader

We developed and evaluated the PiReader, a scanner designed to capture and analyze images of lateral flow-based tests.

#### 1) Hardware

**FIGURE 2.**
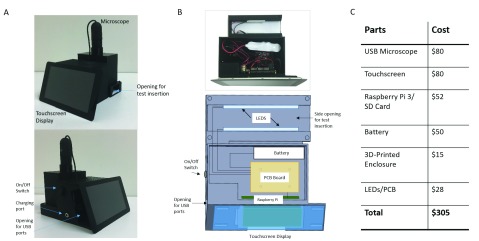
PiReader device: A) labeled device photos, B) (top) photo of inner components with microscope removed, (bottom) labeled diagram layout of inner components, and C) cost of materials.

**Fig. 2** shows a schematic diagram and photos of the PiReader. The PiReader was designed to create a portable imaging system that could scan and interpret HPV vaccination test results at the point-of-care. The system consists of an LED illuminator, a lateral flow test holder, and a USB digital microscope (5MP, interpolated $220\times$ magnification, video rate of 30 FPS), all enclosed in a 3D printed case (roughly $16\times 16\times20$ cm in size). Lateral flow tests are inserted into the enclosed reader and illuminated by two white LED-panel backlights (3V, 20mA). The bottom of the chamber is lined with white paper to provide even background for imaging. The microscope is controlled by a Raspberry Pi v3 single-board computer, which captures and analyzes the images of lateral flow tests. The system is powered by a 6.4V rechargeable battery. Images and results are displayed on a Raspberry Pi 7” touchscreen display. The total cost of material goods for the PiReader is $305.

The PiReader image analysis software was developed in Python and incorporates Open Source Computer Vision Library software (OpenCV). When the PiReader is switched on, the home screen appears and allows the user to select the type of lateral flow test they would like to analyze. Once selected, a live view from the microscope is displayed on the touchscreen, and fiducial marks are displayed to indicate that a test has been properly inserted into the reader. Once the test is in place, the user presses the “Start Analysis” button on the touchscreen. Test results appear on the touchscreen within one second.

#### 2) Image Analysis

**FIGURE 3.**
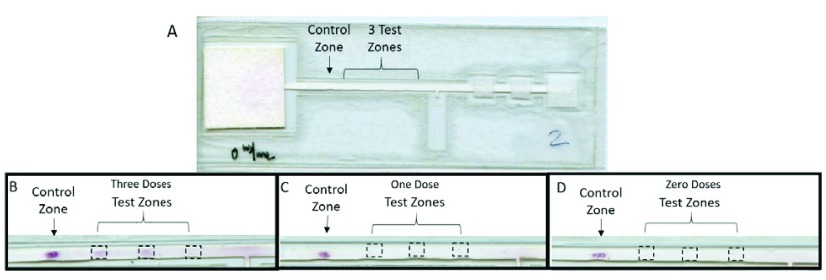
Images of the paper-based HPV vaccination test. A) Image of the HPV vaccination test strip. Size: $5.5 \times 13$ cm. Images of the test after running samples from human subjects who received B) three doses of HPV vaccine, C) one dose of HPV vaccine, and D) zero doses of HPV vaccine.

The PiReader was initially designed to automatically evaluate the results of a lateral flow HPV serology test previously described by Grant et al, **Fig. 3** [30]. The test detects the presence of HPV antibodies in the blood to help health care providers identify whether patients have received two or more doses of the HPV vaccine. This is especially important in low-resource settings where medical records may be hard to obtain or do not exist. The lateral flow test has four readout areas, including a control zone and three HPV antibody capture zones (three test zones). The control zone presents with a positive signal to indicate that the test has run correctly. The test is designed so that the number of positive antibody capture zones correlates to the amount of HPV antibody in a patient’s blood. If a patient received two or more doses of vaccine, then 2–3 of the test zones will present with a positive signal. If the patient has received zero or a single dose of vaccine, then only 0–1 of the test zones are positive.

**FIGURE 4.**
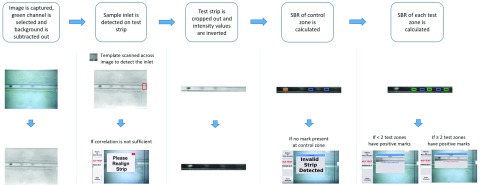
Flow diagram summarizing the image analysis performed by the PiReader with representative images. The intensity value corresponding to the 90th percentile of pixel intensity values of the control zone (orange box) and each of the three test zones (green boxes) are compared to the average background intensity (blue boxes) of the test strip to calculate the signal-to-background ratios (SBRs) for each. If two or more of the test zones have positive marks (SBRs ≥ 1.6), then the tested sample is identified as having received 2–3 doses of HPV vaccine, otherwise the test sample is identified as having received < 2 doses of HPV vaccine.

**Fig. 4** shows the image analysis steps along with example images. Once the user presses “Start Analysis”, the displayed image from the live view is captured and saved for analysis. The green channel is selected and a background image is subtracted out to account for non-uniformities in illumination. Next, feature detection algorithms from OpenCV, are used to find the sample inlet on the test strip. The detected inlet serves as a reference point from which the positions of the control zone and test zones are identified. The intensity value corresponding to the 90th percentile of pixel intensity values within the control zone and test zones are calculated and compared to the average pixel intensity value between the test zones. The signal-to-background ratio (SBR) for each zone is calculated by dividing the signal from the zone by the average background signal. A zone is deemed positive if the recorded SBR for that zone is greater than or equal to the preset SBR threshold. The final results are displayed on the touchscreen display.

All images generated during image analysis are saved onto the Raspberry Pi in a file identified by the date and time of the reading. The final result, along with SBR calculations, are saved in a text file within the same folder.

#### 3) In Vitro Evaluation

To compare performance of the PiReader to a flatbed computer scanner, test strips were prepared using plasma from a non-vaccinated volunteer spiked with different concentrations of HPV16 antibody and scanned using both systems. The concentrations tested were 0, 0.5, 1, 2, 4, 8, 16, and $32~\mu \text{g}$/mL. Each concentration was run on three separate test strips. Each paper strip was read using the original computer scanner described in Grant *et al.* [30] and read three separate times using the PiReader.

According to Villa et al, those naturally infected with HPV exhibit plasma concentrations of anti-HPV16 ranging from 50–100 milli-Merck Units per milliliter (mMU/mL), while those vaccinated against HPV exhibit levels greater than 800 mMU/mL [35]. Opalka et al showed that 50 ng/mL is approximately equal to 4.6 mMU/mL [36]. Using this conversion, it was calculated that individuals previously infected with HPV would be expected to have plasma antibody levels between 0.5-$1.0~\mu \text{g}$/mL, while those fully vaccinated against HPV would be expected to have levels above $8~\mu \text{g}$/mL. Therefore, the test should distinguish samples with anti-HPV16 concentrations of $8~\mu \text{g}$/mL and above (positive for HPV vaccination) from samples with anti-HPV16 concentrations of $1~\mu \text{g}$/mL or less (negative for HPV vaccination). In order to determine the SBR threshold for distinguishing a positive from negative result, the average SBR for a negative control zone and negative test zones were calculated. The final threshold was set three standard deviations above the average SBR for a negative result.

#### 4) Clinical Evaluation

To evaluate the ability of the PiReader to accurately analyze HPV serology tests from patient samples, we used the reader to re-read tests obtained in a previously reported clinical evaluation by Grant *et al.* [30]. Nine tests were read and interpreted by the PiReader three separate times. For comparison, the image analysis described in Grant et al. was performed on the tests again using the same high-resolution computer scanner.

## III. Results

### A. PiHRME

The lightweight CNN model was able to successfully transfer learn the binary output of the existing HRME image analysis algorithm with high accuracy. Initial model accuracy of the MobileNetV2 on the first training/validation batches were 57% and 50% respectively. Upon completion of 4,000 training iterations, the training and validation of the final batches improved to 93% and 92% respectively. Final accuracy for the 405 images in the test set was 90%. Whereas the raw numerical output of the pre-existing algorithm is a metric ranging from 0 to 417 with a positivity threshold of 120, the output of the retrained CNN ranged from 0 to 1 with a binary threshold for positivity of 0.50. In deployment, the CNN required an average of 5.2 seconds to perform inference on a single frame. The frame rate of the PiHRME system was much slower with real-time inference activated (30 FPS vs 0.19 FPS). Although the image processing time was not ideal for real-time inference, it was still faster than the pre-existing algorithm running on a PC tablet which requires ~8 seconds per frame.

#### 1) In Vitro Evaluation

**FIGURE 5.**
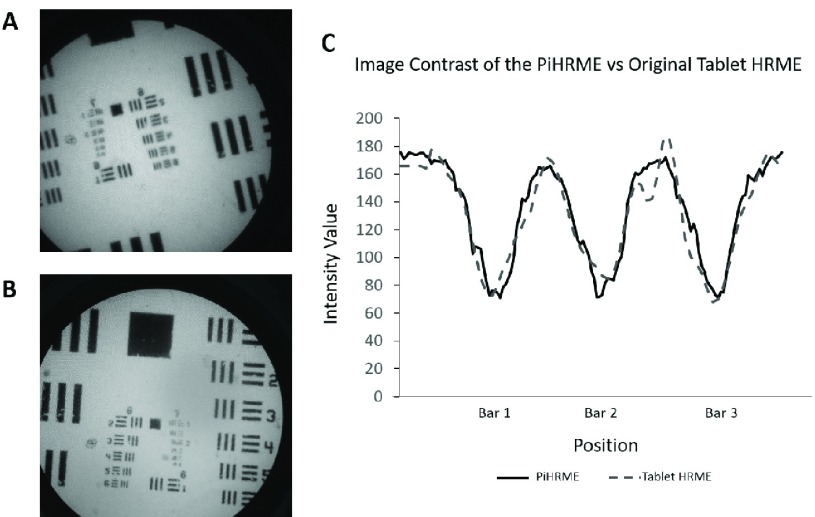
Air Force resolution target images taken using A) the original tablet HRME system and B) the PiHRME. Both have a final resolution of $4.4 ~\mu$m. C) Plot of the intensity values across the three vertical bars of Group 6 Element 1 when using the PiHRME vs the tablet HRME demonstrate similar image contrast.

**Fig. 5** shows images of an Air Force target imaged with both the original tablet-based HRME system (**Fig. 5A**) and PiHRME system (**Fig. 5B**). Both can resolve Group 6 Element 6 of the target, indicating a lateral spatial resolution of $4.4~\mu \text{m}$. **Fig. 5C** shows a plot of the intensity profile across the three vertical bars of Group 6 Element 1 by both the PiHRME and tablet HRME. The similar intensity profiles indicate similar image contrast is achieved by both HRME systems.

**Video 1** shows real-time imaging results obtained from different calibration targets with the PiHRME running the CNN algorithm. The beginning of the video shows a PiHRME image obtained from a calibration target simulating the morphology of high-grade cervical precancer. The CNN algorithm reports a 0.67 probability score, correctly identifying image features that represent high-grade precancer. Next, the video shows images acquired from a calibration target representative of normal cervix. The probability score updates in real-time to 0.22, correctly identifying image features representative of normal epithelium. The probability score continues to update with different image frames, accurately identifying each.

**Video 1.**
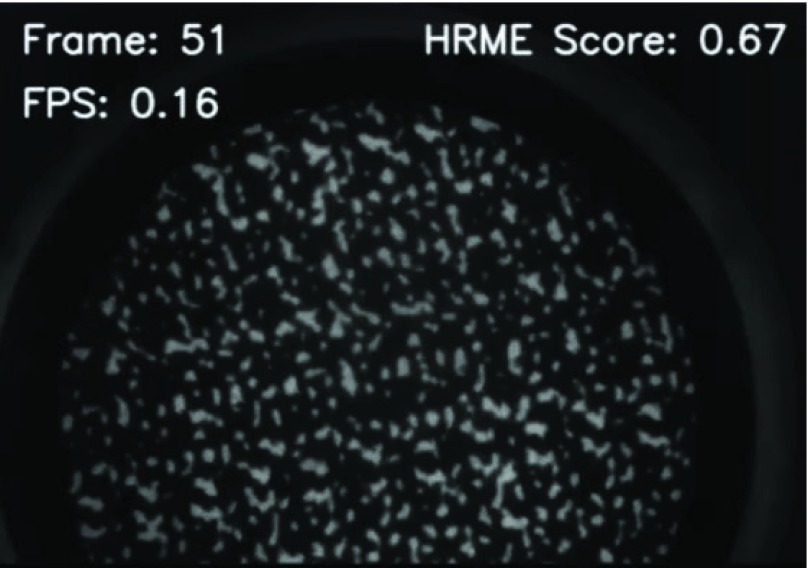
Video of real-time PiHRME imaging of calibration targets. HRME probability score changes in real-time. Scores less than 0.50 indicate that the image is likely representative of normal/benign cervical tissue, while a score of 0.50 and above indicates the image is likely representative of high-grade cervical precancer or cancer.

#### 2) Ex Vivo Evaluation

**FIGURE 6.**
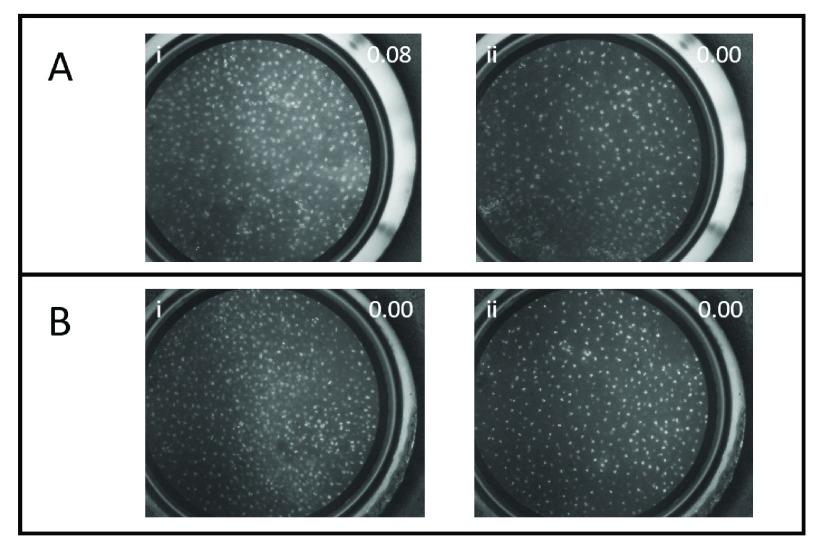
PiHRME images of two LEEP specimens showing the probability score provided by the CNN image analysis algorithm. A) Images taken of LEEP specimen #1 of a visible lesion at i) 6:00 and of a visibly normal area at ii) 8:00. B) Images taken of LEEP specimen #2 of two visibly normal areas at i) 1:00 and at ii) 8:00. Final pathology diagnosis for both LEEP specimens was negative for high-grade precancer(CIN1).

**Fig. 6** shows images from two different LEEP specimens taken with the PiHRME. The first LEEP specimen had one visible lesion at 6:00 by colposcopy, while the second LEEP specimen had no visible lesions. Images of the lesion and one visibly normal area of the first LEEP specimen and images at two visibly normal areas from the second LEEP specimen were collected. All images received scores of < 0.50 using the CNN image analysis algorithm. These results were consistent with the histopathologic diagnosis for both LEEP specimens which were negative for high-grade cervical intraepithelial neoplasia and cancer.

#### 3) In Vivo Evaluation

**FIGURE 7.**
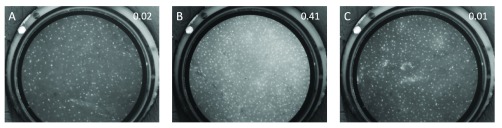
In vivo PiHRME images of the cervix showing the probability score provided by the CNN image analysis algorithm at the top of each image. A & B) Images taken of a cervical lesion suspicious for high-grade precancer at two separate sites. C) Image taken of a visibly normal area of the cervix. All images were categorized by the CNN image analysis algorithm as being of normal/benign tissue. The final pathology diagnosis for this patient was chronic inflammation.

**Fig. 7** shows PiHRME images collected from a woman undergoing a cervical examination for cervical cancer screening and diagnosis in El Salvador. During the examination, a visible lesion was noted by the clinician as concerning for high-grade cervical precancer. PiHRME images were taken of the lesion at two different sites and cervical biopsies were collected. An image was also taken of a visibly normal area. The two images taken of the lesion and the image taken of the visibly normal area all received scores < 0.50: 0.02, 0.41, and 0.01 respectively (all HRME negative). This diagnosis by the PiHRME corresponded with the patient’s final pathologic diagnosis of chronic inflammation.

### B. PiReader

#### 1) In Vitro Evaluation

**FIGURE 8.**
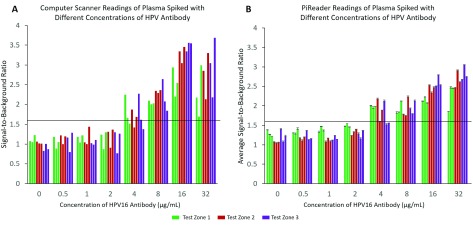
Signal-to-background ratios (SBRs) of plasma spiked with different concentrations of HPV16 antibody read by A) computer scanner and B) PiReader. Each sample was scanned three separate times by the PiReader. The average SBR is shown in (B) with error bars reflecting one standard deviation. The threshold for positivity is a SBR at or above 1.6. The HPV vaccine test was consistently read as positive by both imaging methods when running samples containing HPV antibody concentrations of 8 $\mu$g/mL or more.

**Fig. 8** shows the average SBR readings for plasma samples spiked with different concentrations of anti-HPV16 using the computer scanner and PiReader. The average test zone SBR for the samples containing no anti-HPV16 was 1.20 ± 0.13. Similarly, the average SBR for a negative control zone was found to be 1.16 ± 0.14, therefore the same SBR threshold for positivity was set for both the control and test zones at ≥ 1.6. At this threshold, both devices reliably read all samples with anti-HPV16 concentrations of $8~\mu \text{g}$/mL and above as positive, while all samples with concentrations of 2 $\mu \text{g}$/mL and below are read as negative. This appropriately separates the two groups we want to distinguish, fully vaccinated individuals (anti-HPV16 concentrations of $8~\mu \text{g}$/mL and above) from those not vaccinated or who have been previously infected with HPV (anti-HPV16 concentrations of $1~\mu \text{g}$/mL and below).

#### 2) Clinical Evaluation

**FIGURE 9.**
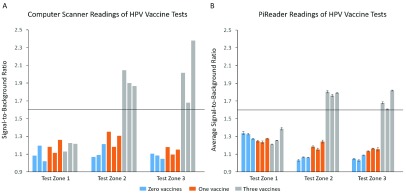
Signal-to-background ratios (SBRs) of HPV vaccine tests read by A) computer scanner vs. B) PiReader. Each test was scanned three separate times by the PiReader. The average SBR is show in (B) with error bars reflecting one standard deviation. The threshold for positivity is a SBR at or above 1.6. Tests contained samples from human subjects where three received zero doses of HPV vaccine, three received one dose of HPV vaccine, and three received three doses of HPV vaccine.

**Fig. 9** shows the SBR readings of the test zones for each of the nine HPV vaccination tests when read using the original high-resolution computer scanner versus the PiReader. The nine tests were performed using blood collected from human subjects in which three were never vaccinated for HPV, three had only received one dose of HPV vaccine, and three had received three doses of HPV vaccine (fully vaccinated). A horizontal line across each graph indicates the threshold for positivity (SBR ≥ 1.6). The dynamic range of SBR is smaller for the PiReader than for the flatbed scanner. Nevertheless, both the computer scanner and PiReader correctly identified the number of positive test zones in all nine tests.

## IV. Conclusion

The work described here demonstrates the utility of single-board computers for developing low-cost, point-of-care technologies for cervical cancer prevention. Using a Raspberry Pi computer, a low-cost HRME system (PiHRME) was developed ($1,226) at half the cost of the current tablet HRME system ($2,450), while still being able to accurately classify and provide high-resolution imaging of the cervix. In addition, a low-cost, portable lateral flow assay reader (PiReader) was developed to read a point-of-care HPV serology test with the same accuracy as a computer and high-resolution scanner.

The value of using single-board computers, such as the Raspberry Pi, to create biomedical devices for point-of-care diagnostics includes their low cost as well as a number of other advantages. Because of their small size and low-power requirements, they allow devices to be portable and battery powered. They can also be programmed with easy-to-use interfaces to automate analysis of results. These advantages are particularly important when developing medical devices for use in low-resource settings, where electrical power is not always available, where devices often must be operated by local medical personnel with limited clinical laboratory expertise, and where there is limited availability of support for preventive or reparative maintenance. Larger scale clinical field studies are needed to verify these advantages for the PiHRME and PiReader; however, the results presented here indicate that these technologies have the potential to meet the cost, usability, and effectiveness constraints associated with low-resource settings.

The PiHRME was able to provide real-time visualization of cervical nuclei and accurately classify images, which was confirmed by the final histopathology diagnosis. Real-time image analysis when examining the cervix can help clinicians either reject or confirm suspected cases of high-grade precancer or cancer, especially in low-resource areas where expert physicians may not always be available. In order to accomplish real-time image classification for the PiHRME system we leveraged recently developed TensorFlow libraries for the Raspberry Pi (MobileNetV2). Future work will explore using more efficient processors for accelerated neural network computation on embedded systems in addition to other state of the art deep learning models for semantic segmentation and classification using nuclear features. Additionally, the usability and frame-rate could be improved by automatically rejecting low-quality frames (frames with motion blur or not in contact with tissue), and only running inference on high-quality frames.

Ongoing development of TensorFlow Lite can potentially extend this approach even more broadly to microcontrollers and edge computing devices [37]. Image classification using this low-cost ARM architecture was achieved in ~5–6 seconds per frame. The time for analysis can be significantly reduced using hardware acceleration for TensorFlow on field-programmable gate arrays (FPGAs), graphics processing units (GPUs), tensor processing units (TPUs), and Intel processors [38]–[40].

The PiReader demonstrates how real-time imaging can help make lateral flow test interpretation easier to perform at the point-of-care. The PiReader was designed using a low-cost USB microscope with lower resolution (96 dpi) compared to a commercial flatbed scanner (800 dpi). However, even with lower resolution, there was enough detail within the images to accurately determine the presence and absence of positive signals. We demonstrated the accuracy of the PiReader by interpreting the results of an HPV serology test. The algorithms used here can be applied to read other lateral flow tests that rely on colorimetric changes to distinguish positive and negative results.

Further clinical work is ongoing to validate the utility of these technologies for cervical cancer prevention in low-resource settings. Taken together this work demonstrates the great promise single-board computers have for the development of low-cost, point-of-care medical technologies.
